# Effect of home visits by community health workers on complementary feeding practices among caregivers of children aged 6–23 months in 10 districts of Bangladesh

**DOI:** 10.3389/fpubh.2022.1014281

**Published:** 2023-01-19

**Authors:** Mahfuzur Rahman, Md. Tariqujjaman, Tahmeed Ahmed, Haribondhu Sarma

**Affiliations:** ^1^International Centre for Diarrhoeal Disease Research, Bangladesh (icddr, b), Dhaka, Bangladesh; ^2^National Centre for Epidemiology and Population Health, The Australian National University, Canberra, ACT, Australia

**Keywords:** complementary feeding, children, Bangladesh, community volunteer, promotional intervention

## Abstract

**Background:**

Suboptimal complementary feeding contributes to undernutrition in children aged 6–23 months in low- and middle-income countries like Bangladesh. Multifaceted interventions have been taken to improve complementary feeding practices, but there is limited evidence about the effect of home visits by community health workers (CHWs) on complementary feeding practices of the caregivers of children.

**Methods:**

We collated data from the baseline and the corresponding follow-up survey done as part of an evaluation of Bangladesh Maternal, Infant and Young Child Nutrition (MIYCN) programme. We collected data on complementary feeding practices using a 24-h recall questionnaire. Age-appropriate dietary diversity, minimum meal frequency, and minimum acceptable diet were assessed based on recommended food consumption as per child's age. To understand the effect of CHWs' visits on complementary feeding practices, we performed Generalized Estimating Equation (GEE) procedure for dealing with correlated data and adjusted other covariates.

**Results:**

A total of 758 and 745 caregivers of children aged 6–23 months participated in the baseline and follow-up survey, respectively. Complementary feeding practices were improved in 1 year of programme implementation; dietary diversity increased from 46 to 54%, minimum meal frequency from 82 to 91%, and minimum acceptable diet from 41 to 53%. Caregivers of children who had received the visit of CHWs in the last 12 months prior to the day of the follow-up survey were more likely (AOR 1.51; 95% CI 1.10–2.10) to maintain dietary diversity in their children's feeding practices than the caregivers who had not received a CHW visit in the last 12 months. The likelihood of maintaining a minimum acceptable diet in feeding practices was 1.57 times higher (AOR 1.57; 95% CI 1.14–2.17) among the caregivers who were exposed to the visits of the CHWs in the last 12 months compared to the caregivers who were not exposed to the CHW's visits in the last 12 months.

**Conclusion:**

Integration of promotional activities of complementary feeding practices into the mainstream nutrition programme can be instrumental in optimizing complementary feeding practices among the caregivers of the under-five children. However, home visits by CHWs should be prioritized in such an integrated programme.

## Introduction

Globally, the prevalence of undernutrition among preschool children is high. Among the under-five children in Bangladesh, 31% are stunted, 8% are wasted, and 22% are underweight (1). Evidence shows that different interventions have been undertaken, and improving complementary feeding practices reduces undernutrition among children (2). However, despite some effective interventions, 35% of children aged 6 to 23 months in Bangladesh are fed a minimum acceptable diet (1). The target of increasing the proportion of children receiving a minimum acceptable diet in accordance with the 4^th^ Health, Population and Nutrition Sector Programme is unlikely to be met with this steady progress (3) Home visits by CHW are instrumental in improving maternal and child health (4–6). It is also evident that home visit by CHW has enhanced the coverage of the programme aimed at preventing micronutrient deficiency among under-five children (7), and CHW's performance has an effect on the behavior of the caregivers of children (8). However, there is limited evidence of the effect of home visits by CHWs on complementary feeding practices of caregivers when the infant and young child feeding (IYCF) programme is integrated into the mainstream nutrition programme.

When the programmes are integrated, the expected outcomes of the programmes are more difficult to explain. For instance, prevention of micronutrient deficiency as a stand-alone programme has not been shown to reduce undernutrition, but when such a programme is integrated with IYCF programme, reducing undernutrition appears as an outcome (9). Nevertheless, an intervention intending to the prevention of micronutrient deficiency among children does not undermine complementary feeding practices; rather, exposure of beneficiaries to home visits by CHWs- crucial activities of the integrated programme may have a positive association with improved complementary feeding practices (10).

BRAC, a non-governmental organization, has implemented the Maternal, Infant and Young Child Nutrition (MIYCN) Phase II programme in Bangladesh in partnership with the Global Alliance for Improved Nutrition (GAIN), Renata Pharmaceutical Company (manufacturer of MNP) and Social Marketing Company (SMC) (11). BRAC has implemented this MNP programme in integration with its mainstream Health, Nutrition and Population (HNP) programme through the network of *Shasthya Shebikas* (female volunteer frontline community health workers). These volunteer community health workers (CHWs) are the first point of contact for the community people receiving healthcare services from BRAC. BRAC is distributing Pushtikona-5 (a brand name of BRAC's MNP) sachets containing Iron, vitamin A, vitamin C, folic acid, and zinc through their *Shasthya Shebikas* in the community at a subsidized price. Although BRAC started distributing Pushtikona-5 as part of their mainstream HNP in 2010, the MIYCN Phase II programme emphasized on its promotional activities, such as training for the health workers to promote and supply the product to them, to reduce anemia among the children aged 6–59 months. The training was not limited to promoting the product; it also covered the contents on how to counsel the caregivers to improve complementary feeding to the children.

The icddr,b- a Bangladesh-based international health research institute was commissioned to evaluate this MIYCN programme, and it conducted cross-sectional surveys as part of periodic assessment of the programme. A detailed description of the evaluation and BRAC programme is published elsewhere (7, 12, 13). For this article, we used data from two cross-sectional surveys (a baseline and a follow-up survey) conducted in 10 districts of the programme areas to understand the effect of home visits by CHWs on complementary feeding practices among caregivers of the children aged 6–23 months. Prior to the rollout of the interventions under the MIYCN program, BRAC's maternal, neonatal, and child health (MNCH) program was in place in these 10 districts. The MNCH programme's activities included providing basic primary healthcare at the community level, working with village health committees to motivate behavior change in the community by addressing the issues of pregnancy, newborn and child health, and facilitating access to obstetric and newborn care at both public and private facilities (14). As part of MNCH programme, the trained CHWs made doo-to-door visits in their catchment areas, offered essential healthcare services and disseminated messages on maternal and child nutrition (13).

Although there are some evidences that nutrition education intervention for caregivers of the children can improve their complementary feeding practices (15), there is a paucity of findings about the effect of home visits by CHWs on complementary feeding practices when the infant and young child feeding (IYCF) programme is integrated into the mainstream nutrition programme. In this study, we intended to understand the effect of home visits by CHWs on complementary feeding practices among the caregivers of children when the programme is integrated into a mainstream nutrition programme. It is anticipated that the results described in this paper will provide directives for programme implementers in determining a comprehensive outcome of the programme while integrating complementary feeding programme with mainstream health, population and nutrition programmes. It is also expected that the findings of the study will provide policymakers with insights into how to effectively involve CHWs in improving complementary feeding practices among caregivers of children aged 6–23 months at the national level.

## Methods

### Data source

We used data from two cross-sectional surveys (baseline and the corresponding follow-up survey after 1 year) that were conducted as part of an evaluation of the Bangladesh MIYCN programme. Although during the baseline and the corresponding follow-up survey, data were collected from caregivers of children aged 6–59 months, we extracted data on caregivers of children aged 6–23 months for this study.

### Study population

Caregivers of children aged 6–23 months were the study population in this study. A caregiver was defined as the child's biological mother or the person who took care or looked after and gave the child most meals on most days.

### Sample size and sampling

In the evaluation of MIYCN programme, we estimated sample size considering a 50% prevalence of MNP coverage, a precision of ±10% and a design effect of 2. Thus, our estimated sample size for caregivers of 6–59 months children was 192 households per district.

We performed a two-stage sampling procedure for selecting study participants at the household level. In the first stage, we selected communities or primary sampling units (PSUs) using systematic random sampling, with an equal selection probability for each community or PSU.

Systematic samples of 16 PSUs were drawn from a complete list of the targeted communities or PSUs, which were sorted by district and by sub-district within the programme district to reach the minimum estimated sample size. This ensured that all of the target communities had an equal chance of being selected for the sample. In the second stage, a physical map-segment sample approach was exercised to segment the selected communities or PSUs. Then, the Expanded Programme on Immunization method was applied by spinning a bottle/pen placed in the center of the segment, counting the households along that route and picking the fifth household. The selection of households depended on the inclusion criteria (caregivers of 6–23-months children, mothers/caregivers having resided there for at least 12 months before the day of the interview, the child not being physically challenged or ill).

### Data collection and extraction

**Figure 1:**
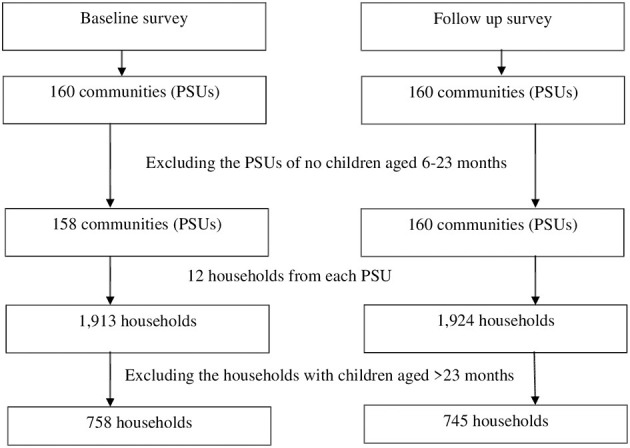
Selection of the households with children aged 6–23 months.

Once a caregiver was selected for data collection, a well-informed written consent was taken from her before commencing the interview. The consent form was written in Bangla language so that the participants could easily understand the objective of the study. The consent form was read out to the caregivers who was unable to read. Signed consent or the left thumb impression was obtained from a caregiver if she agreed to participate in the study. A structured questionnaire was developed keeping the study objectives in mind. Data on complementary feeding practices were collected using a 24-h recall questionnaire. In the 24-h recall method, we asked the caregivers of the children what foods including drinks and beverages they fed the index children in the last 24 h. In addition, the questionnaire captured data on other demographic and socio-economic status including age of the participants and index children, number of household members, education and occupation of the parents of index children, religion and wealth status of the households. We asked the caregivers of the children if they received any visits by a CHW in the last 12 months prior to the day of survey to assess their exposure to CHW visits. Interviews with the caregivers of children were conducted at the household level, and data were collected using an electronic device: a smartphone with the application of Open Data Kit (ODK). The baseline and the corresponding follow-up survey were done in 10 districts of Bangladesh in 2014 and 2015 respectively. A total of 160 communities or PSUs where BRAC's MIYCN programme was being implemented with the involvement of *Shasthya Shebika*, were sampled from the 10 districts in each survey at the first stage of sampling. To reach the representative sample size, 12 eligible households with at least a child of 6–59 months were selected. In this study, we used data of households with children aged 6–23 months and so, at first, we excluded the PSUs where there were no children aged 6–23 months. Thus, 2 PSUs were excluded from the baseline survey. Finally, from the remaining PSUs (158 at baseline and 160 at follow-up survey), we selected the households of children aged 6–23 months (Figure 1).

### Outcome measures

Complementary feeding practice for children aged 6–23 months was the outcome of interest. We measured dietary diversity, minimum meal frequency, and minimum acceptable diet to assess complementary feeding practices. Dietary diversity was assessed based on the consumption of ≥4 food-groups of the children, and minimum meal frequency and minimum acceptable diet were assessed based on the recommended food consumption, by the World Health Organization as per child's age (16). Table 1 shows the different food-groups with corresponding food items.

**Table 1:** Food-groups and items.

**Food-group**	**Item**
Group-1	Milk and milk products
Group-2	Grains and roots/tubers
Group-3	Vitamin A-rich fruits and vegetables
Group-4	Other fruits and vegetables
Group-5	Pulses
Group-6	Meat and fish
Group-7	Eggs

Demographic and socioeconomic variables were also assessed. We measured the proportion of poor, middle and rich households by wealth index.

### Exposure to home visit by CHW

We defined caregiver's exposure to home visit by CHW as receiving at least one visit by a caregiver from the CHW of BRAC during 1 year of the MIYCN programme intervention. Along with other interventions of health, population and nutrition programme of BRAC, interventions under MIYCN programme were also being implemented with the network of CHWs. Therefore, caregivers who received the visit of CHWs in the last 12 months prior to the day of survey were considered to be exposed to the visit of CHW.

### Data analysis

Descriptive analyses were carried out to assess the characteristics, prevalence and patterns of the study sample and nature of variables. The probability weighting technique was applied to estimate the proportion and mean of the variables of interest. Bivariate analysis including adjusted Wald test and *t*-test were performed to see the changes in the different characteristics between baseline and follow-up period. To understand the effect of a home visit by CHWs on complementary feeding practices, exposure variable (receiving the visit of CHWs in the last 12 months) was kept, along with other potential covariates and confounding factors including child's sex and age, number of household members, household's wealth status and parental education for adjusting in the multiple regression model. We performed Generalized Estimating Equation (GEE) using binary logistic regression models for dealing with correlated data because the data were obtained from different communities or PSUs. We checked multicollinearity among independents variables and found the average variance inflation factor 1.06 indicating negligible collinearity. Data analysis was performed using the statistical software package STATA, version 13 (Stata Crop. 4905 Lake way Drive, College Station, Texas 77845, USA).

### Ethical considerations

The study protocol was reviewed and approved by the Institutional Review Board (IRB) of icddr,b which comprises a Research Review Committee (RRC) and an Ethical Review Committee (ERC). Before conducting the interviews, well-informed written consents were taken from the women who were surveyed.

## Results

### Demographic characteristics of the study participants

A total of 758 and 745 caregivers of children aged 6–23 months participated in the baseline and follow-up surveys respectively. We did not find any significant differences among participants in the baseline and follow-up survey in terms of their household size, their children's age and sex, their education and religious status. However, there was a significant difference in the mean age of the caregivers, from baseline (24.7 ± 5.2) to follow-up survey (25.6 ± 6.1) and the percentage of poor and rich households based on wealth index between baseline (poor: 42.9%, rich: 26.0%) and the corresponding follow-up survey (poor: 27.5%, rich: 39.2%) (Table 2).

**Table 2:** Socio-demographic characteristics of the study participants.

**Variable**	**Baseline (*n* = 758)**	**95% CI**	**Follow-up survey (*n* = 745)**	**95% CI**	***P*-value**
**Household level**					
No. of members in household, (Mean ±SD)	5.2 ± 2.0	5.0–5.3	5.2 ± 2.0	5.1–5.4	0.681
**Caregiver**					
Age (in years) of caregiver, (Mean±SD)	24.7 ± 5.2	24.3–25.0	25.6 ± 6.1	25.1–26.0	0.002
**Father**					
Age (in years), (Mean±SD)	31.4 ± 6.5	30.9–31.8	32.1 ± 6.7	31.6–32.6	0.029
Parental education (≥ 5 years of schooling) %	48.8	45.3–52.4	52.0	48.3–55.5	0.215^¶^
**Children**					
Age in months, (Mean±SD)	14.8 ± 5.2	14.4–15.2	14.7 ± 5.2	14.3–15.1	0.640
06–11, % (*n*)	33.9 (257)	30.6–37.4	32.2 (247)	29.9–36.6	0.484^¶^
12–17, % (*n*)	29.3 (222)	26.1–32.6	30.7 (229)	27.5–34.2	0.554^¶^
18–23, % (*n*)	36.8 (279)	33.4–40.3	36.1 (269)	32.7–39.6	0.778^¶^
**Sex of children**					
Female, % (*n*)	45.4 (344)	41.9–49.0	49.4 (368)	45.8–53.0	0.121^¶^
Male, % (*n*)	54.6 (414)	51.0–58.1	50.6 (377)	47.0–54.2	0.121^¶^
**Religion of respondents**					
Hindu or others, % (*n*)	12.8 (97)	10.6–15.4	10.3 (77)	8.3–12.7	0.130^¶^
Muslim, % (*n*)	87.2 (661)	84.6–89.4	89.7 (668)	87.3–91.7	0.130^¶^
**Wealth index**					
Poor, % (*n*)	42.9 (325)	39.4–46.4	27.5 (205)	24.4–30.8	0.000^¶^
Middle, % (*n*)	31.1 (236)	27.9–34.5	33.3 (248)	30.0–36.8	0.361^¶^
Rich, % (*n*)	26.0 (197)	23.0–29.2	39.2 (292)	35.7–42.8	0.000^¶^
SS visit within 12 months of survey, Yes, % (*n*)	67.5 (507)	62.3–72.2	61.0 (448)	55.8–65.9	0.036^¶^

^¶^*P*-values were generated from adjusted Wald test, otherwise t-test.

### Complementary feeding practices among caregivers of children aged 6-23 months

**Figure 2:**
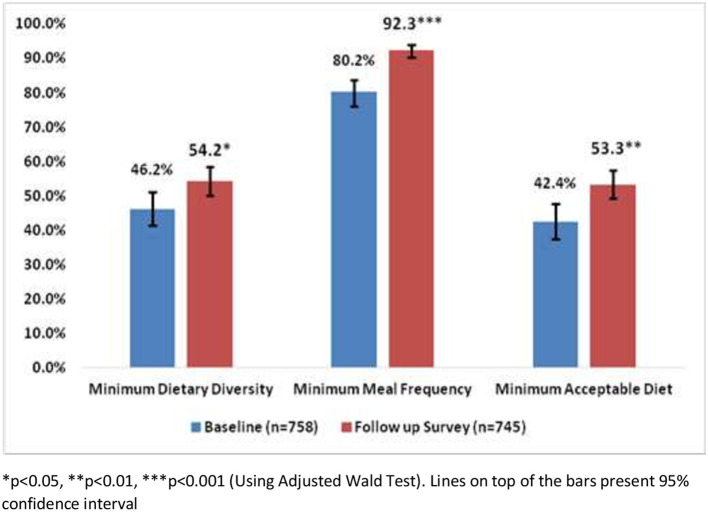
Complementary feeding practices among the caregivers of children aged 6–23 months.

Results showed that complementary feeding practices were improved in 1 year of MIYCN programme implementation (Figure 2). About 46% of the caregivers of children maintained age-appropriate dietary diversity in the baseline survey but, after 1 year of the intervention, 54% of them maintained this. Practices of minimum meal frequency were improved by around 10 percentage points among the caregivers during the intervention period. Results also showed that minimum acceptable diet was maintained by 41% of the caregivers of children in the baseline survey whereas it was maintained by 53% of the caregivers of children in the follow-up survey.

### Complementary feeding practices in the exposed and unexposed groups

**Figure 3:**
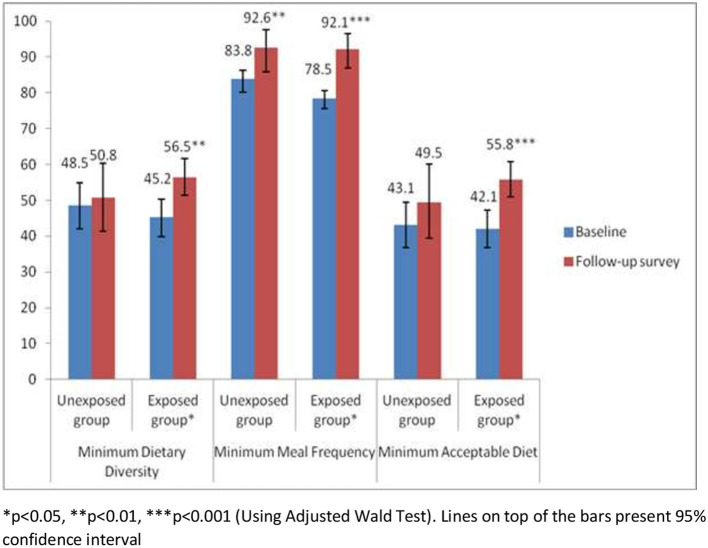
Complementary feeding practices among the caregivers of children by exposed and unexposed groups.

Figure 3 shows that there was no significant change in complementary feeding practices from baseline to follow-up survey among the caregivers who were not exposed to the home visit by CHWs. However, results showed that complementary feeding practices significantly improved from baseline to follow-up survey among the caregivers who were exposed to the home visit by CHWs. Among the exposed group, minimum dietary diversity increased from 45.2% at baseline to 56.5% at follow-up survey. Minimum meal frequency improved from 78.5% at baseline to 92.1% at follow-up survey, and minimum acceptable diet increased from 42.1% at baseline to 55.8% at follow-up survey.

### Association of home visits by CHW with complementary feeding practices

To understand the effect of home visits by CHW on complementary feeding practices, we applied the Generalized Estimating Equation procedure. After adjusting the covariates, results from the multivariable GEE model showed that, during the follow-up survey, dietary diversity and minimum acceptable diet were significantly associated with the CHWs' visits in the last 12 months (Table 3). Caregivers of the children who received the visit of CHWs in the last12 months prior to the day of follow-up survey were more likely (AOR 1.51; 95% CI 1.10–2.10) to maintain dietary diversity in the feeding practices of their children compared to the caregivers who were not exposed to the visit of the CHWs in the last 12 months. The likelihood of maintaining minimum acceptable diet in the feeding practices was 1.57 times higher (AOR 1.57; 95% CI 1.14–2.17) among the caregivers who were exposed to the visit of the CHWs in the last 12 months compared to the caregivers who were not exposed to the CHW's visit in the last 12 months prior to the day of follow-up survey.

**Table 3:** Association of exposure variable with complementary feeding practices.

**Variable**	**Baseline**		**Follow-up survey**	
	**AOR** ^†^	**95% CI**	**AOR** ^†^	**95% CI**
**Dietary diversity (outcome)**				
Visit of the CHWs in the last 12 months (Ref: Received no visit)	1		1	
Received visit	1.33	0.95–1.88	1.51^*^	1.10–2.10
**Minimum meal frequency (outcome)**				
Visit of the CHWs in the last 12 months (Ref: Received no visit)	1		1	
Received visit	1.06	0.72–1.57	1.37	0.80–2.34
**Minimum acceptable diet (outcome)**				
Visit of the CHWs in the last 12 months (Ref: Received no visit)	1		1	
Received visit	1.37	0.98–1.94	1.57^**^	1.14–2.17

^*^P < 0.05; ^**^P < 0.01; AOR, Adjusted odds ratio; CI, Confidence interval. ^†^Adjusted for child's sex and age, household size, household wealth status and parental education.

## Discussion

Results of this study clearly demonstrated the positive effect of CHWs visits on complementary feeding practices when infant and young child nutrition program is integrated into a mainstream nutrition programme. This finding corresponds with the evidences generated from other study that also suggests that a mainstream maternal and child care programme could be a viable avenue to improve acceptance and utilization of other services pertaining to child nutrition (17). Overall complementary feeding practices were improved from baseline to follow-up survey in 10 rural districts of Bangladesh where the MIYCN programme activities were being implemented in integration with BRAC's maternal, neonatal and child health (MNCH) care services under a mainstream health, nutrition and population programme (HNPP). Results of this study clearly demonstrated that the caregivers who were exposed to home visit by CHWs had improved complementary feeding practices during the intervention period. Findings from this study substantiate the notion that complementary feeding practices among the caregivers of under-five children can be promoted in an integrated mainstream nutrition programme where the home visit of CHW is considered as pivotal. However, home visit by CHW can be influenced by programmatic determinants such as incentives and other non-financial motivations (18, 19) and thus programme might have unintended consequences on its outcomes (13).

Although this study was not intended to identify the factors associated with home visit of CHW for promoting complementary feeding practices among the caregivers of under-five children, the findings of the study reinforce the necessity of home visit by CHW. Therefore, while designing a programme aiming at promoting complementary feeding practices it is imperative to take the aspects into account that can increase home visit by CHW. Training is one of the vital aspects that can improve self-efficacy and self-confidence of a CHW and thus, encourages her or him to visit the households of the caregivers of children.

When a CHW works in a mainstream nutrition programme integrated with promotional programme of complementary feeding practices, the CHW is very likely to receive intensive training under different components of the programme. The application of knowledge and skills that the CHW acquire from the training arranged under different components of the programme also can result in improving complementary feeding practices of the caregivers of children served by them (20).

Results of this study showed that the caregivers of the under-five children who were exposed to the home visits by CHWs maintained more complementary feeding practices compared to the caregivers who were not exposed. It indicates that counseling by the CHW during home visit works. Nevertheless, the caregivers of the children are more likely to listen to those CHWs who are acceptable to them. Evidence shows that acceptancy of the CHWs depends on how they are selected, trained and supervised (21). Another study indicates that in addition to adequate training the implementers should be cautious about the workload of the CHWs (22). Given that their engagement in the programme is voluntary, burden of their workload can demotivate them and thus, can result in their substandard performance. Therefore, CHWs should get support from progrmme perspective in terms of task distribution when they get involved in an integrated programme with several components. The CHWs should get support of health systems as well in choosing the best technical interventions for them as well as in supplying the necessary training, supervision, and logistical support for these programmes (23). Therefore, programmes that intend to promote complementary feeding practices among the caregivers of under-five children through CHWs should take different strategies into consideration at the initial stage. A comprehensive implementation research approach would be worthwhile to develop the strategies that can promote the performance of CHWs (24).

Although complementary feeding practices among the caregivers of children have been improved during the intervention period, this result should be interpreted cautiously since there might have secular trend of improving complementary feeding practices among the caregivers in the intervention areas. It could otherwise overestimate the effect of home visit by CHW on complementary feeding practices.

### Study limitations

This study was cross-sectional in design and, so, it could not show any causal relationship of home visit by the CHW with complementary feeding practices. Data were collected from 10 districts of Bangladesh, so it may not necessarily be representative of other districts of the country where BRAC's MIYCN programme was not implemented. In addition, the individuals in the baseline and follow-up samples were not the same. Therefore, the results may not represent the actual effect of home visits by the CHW on complementary feeding practices among the caregivers of the children.

## Conclusion

Positive association of home visits by CHWs with complementary feeding practices generated an evidence-base for the integration of complementary feeding promotional programme into the mainstream maternal, neonatal and child health programme for achieving comprehensive outcomes, like optimal IYCF practices among caregivers of under-five children. Integration of promotional activities of complementary feeding practices with the mainstream nutrition programme can be instrumental in optimizing complementary feeding practices. However, home visit by CHWs should be prioritized in such an integrated programme.

## Data availability statement

The raw data supporting the conclusions of this article will be made available by the authors, without undue reservation.

## Ethics statement

The studies involving human participants were reviewed and approved by the Institutional Review Board (IRB) of icddr,b. The patients/participants provided their written informed consent to participate in this study.

## Author contributions

MR contributed to conceptualization of the study and drafted the manuscript. MR and MT analyzed data. HS acquired funding for this study. MT, HS, and TA reviewed and edited the manuscript. All authors critically revised the manuscript, agree to be fully accountable for ensuring the integrity and accuracy of the work, read, and approved the final manuscript.
